# Supplementary material for: Pre-Slavic and Slavic Interaction at Eastern Periphery of Slavic Expansion in Northeastern Europe (Y-Gene Pools of Volga-Oka Region)
Source: Genes (Basel). 2025 Sep 27;16(10):1149. doi: 10.3390/genes16101149 (PMC12562464; doi:10.3390/genes16101149)
Supplement: Supplementary file 1 [file genes-16-01149-s001.zip › Supplementary Figures S1-S5. Phylogenetic search networks.pdf]

Supplementary Figures S1-S5. Phylogenetic search networks

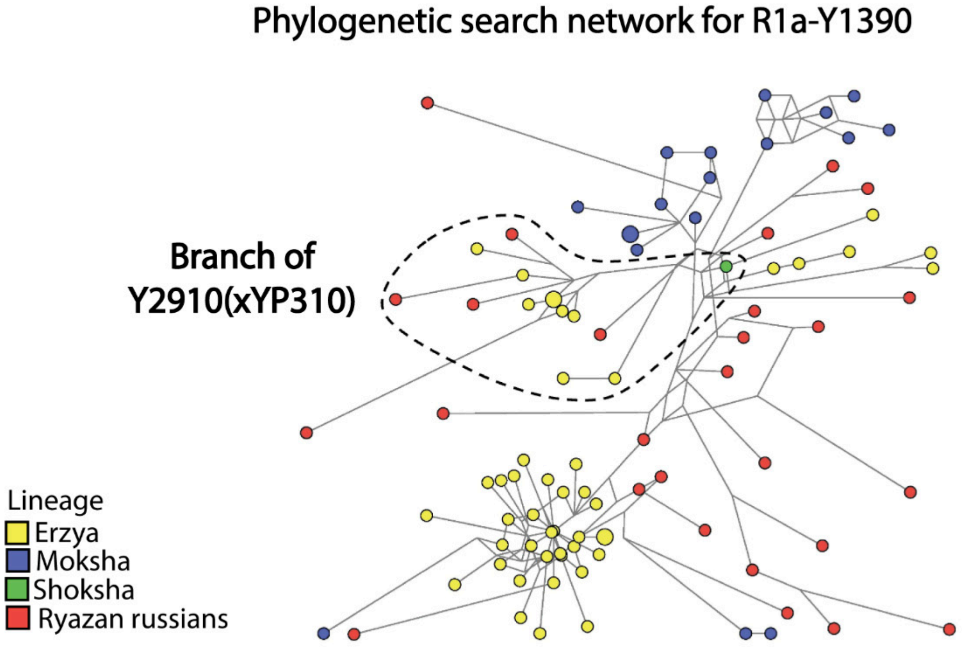

Supplementary Figure S1. Y-STR phylogenetic network for the branch R1a-Y1390 to search clusters of probable pre-Slavic population descendants.

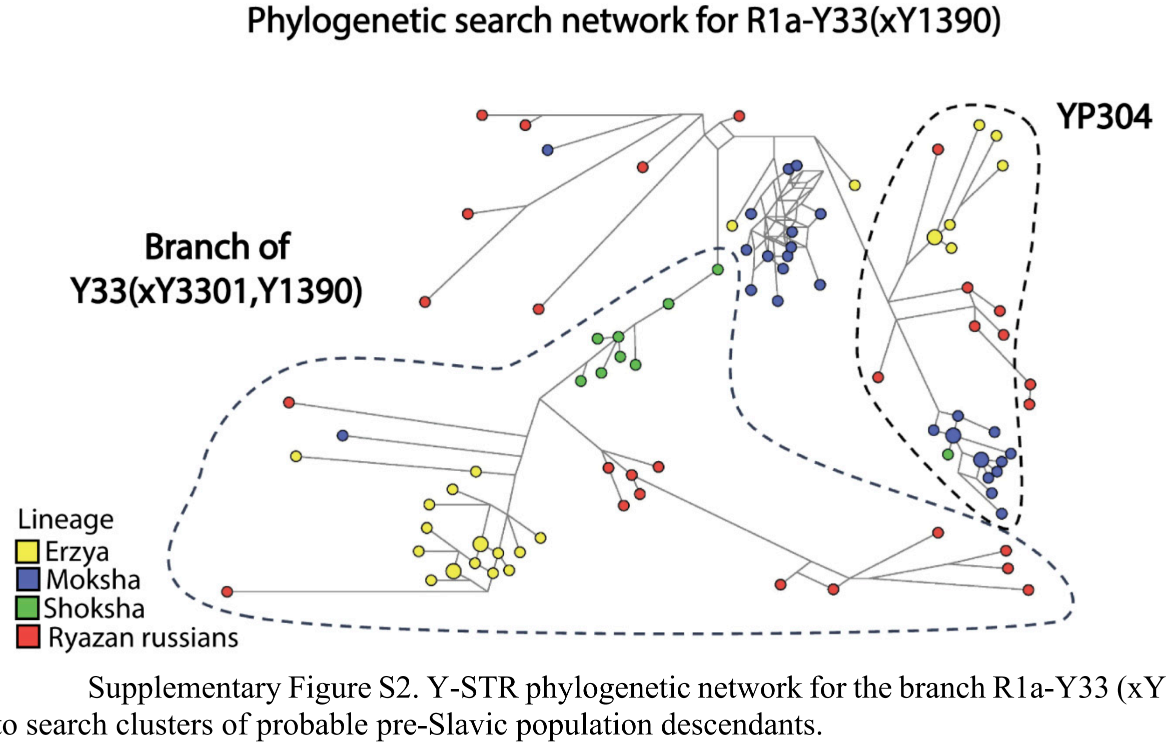

Supplementary Figure S2. Y-STR phylogenetic network for the branch R1a-Y33 (xY1390) to search clusters of probable pre-Slavic population descendants.

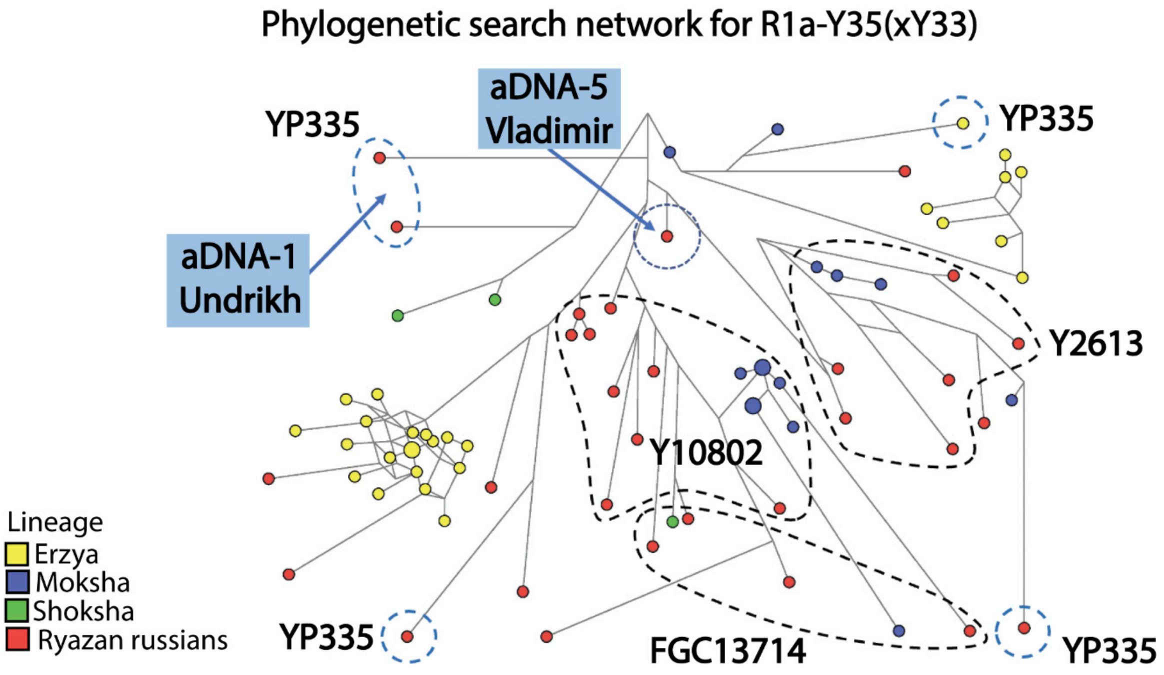

Supplementary Figure S3. Y-STR phylogenetic network for the branch R1a-Y35 (xY33) to search clusters of probable pre-Slavic population descendants.

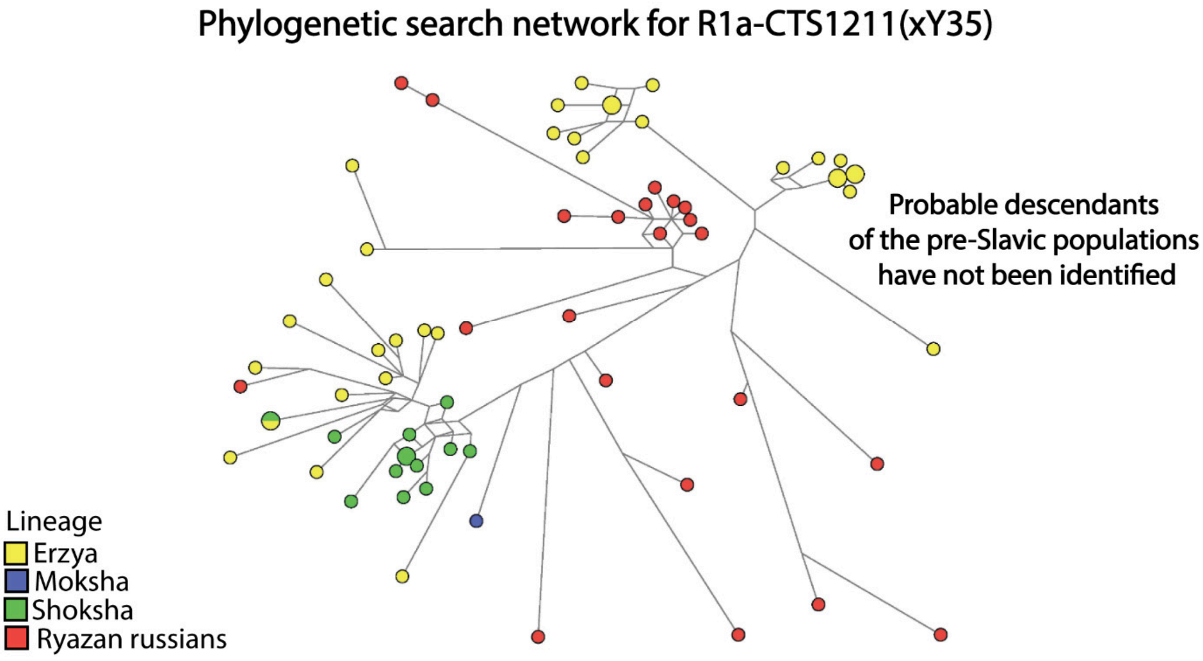

Supplementary Figure S4. Y-STR phylogenetic network for the branch R1a-CTS1211 (xY35) to search clusters of probable pre-Slavic population descendants.

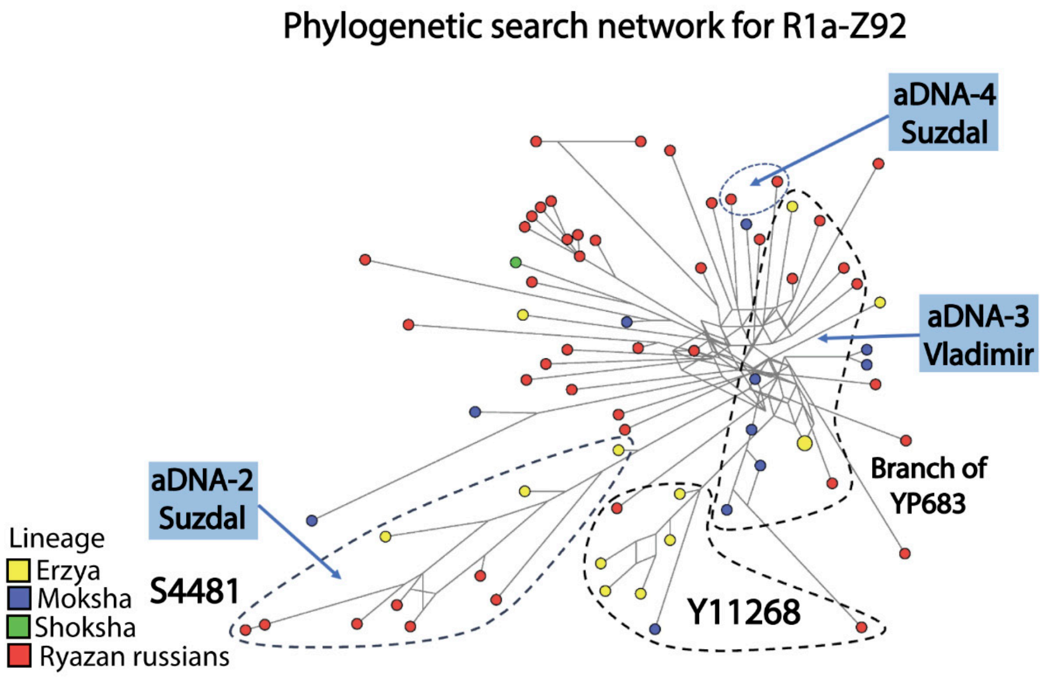

Supplementary Figure S5. Y-STR phylogenetic network for the branch R1a-Z92 to search clusters of probable pre-Slavic population descendants.
