# Supplementary material for: Pre-Slavic and Slavic Interaction at Eastern Periphery of Slavic Expansion in Northeastern Europe (Y-Gene Pools of Volga-Oka Region)
Source: Genes (Basel). 2025 Sep 27;16(10):1149. doi: 10.3390/genes16101149 (PMC12562464; doi:10.3390/genes16101149)
Supplement: Supplementary file 1 [file genes-16-01149-s001.zip › Supplementary Figures S6-S15. Phylogenetic search trees.pdf]

Supplementary Figures S6-S15. Phylogenetic search trees

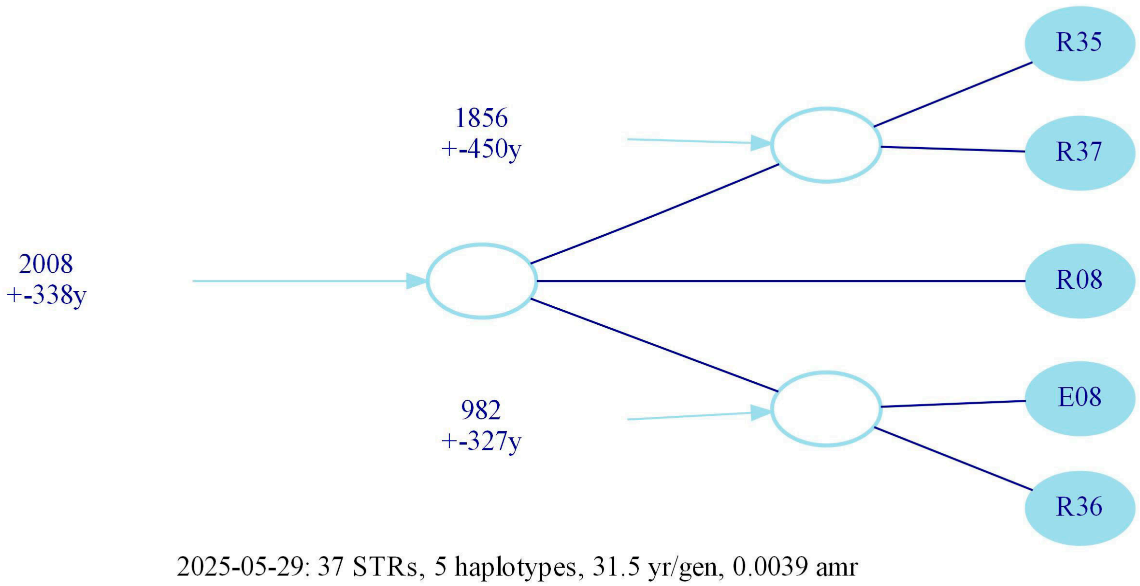

Supplementary Figure S6. Phylogenetic search tree for the branch R1a-YP335 to confirm pre-Slavic origin of found Y-STR cluster. Designations of samples: R – Ryazan Russians; E – Erzya.

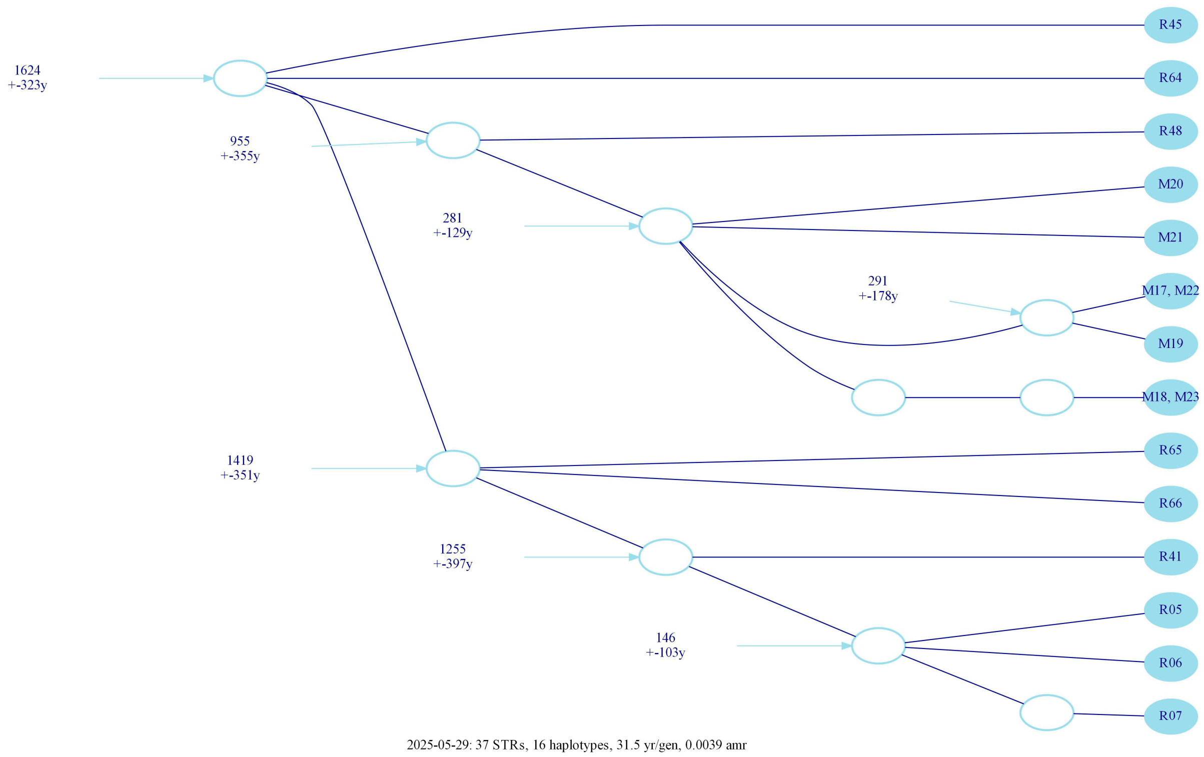

Supplementary Figure S7. Phylogenetic search tree for the branch R1a-Y10802 to confirm pre-Slavic origin of found Y-STR cluster. Designations of samples: R – Ryazan Russians; M – Moksha.

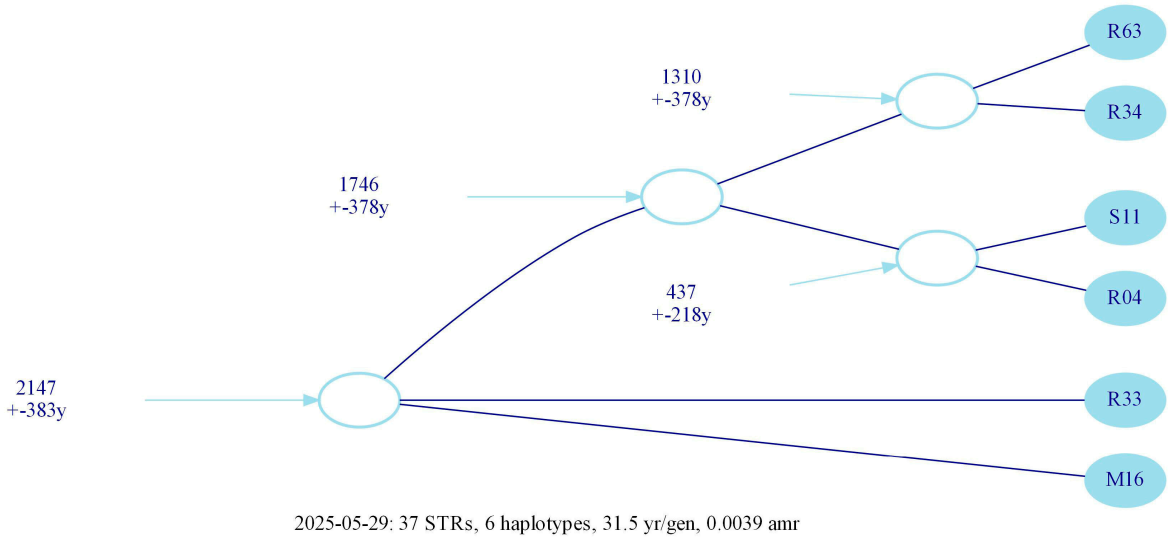

Supplementary Figure S8. Phylogenetic search tree for the branch R1a-FGC13714* to confirm pre-Slavic origin of found Y-STR cluster. Designations of samples: R – Ryazan Russians; M – Moksha; S – Shoksha.

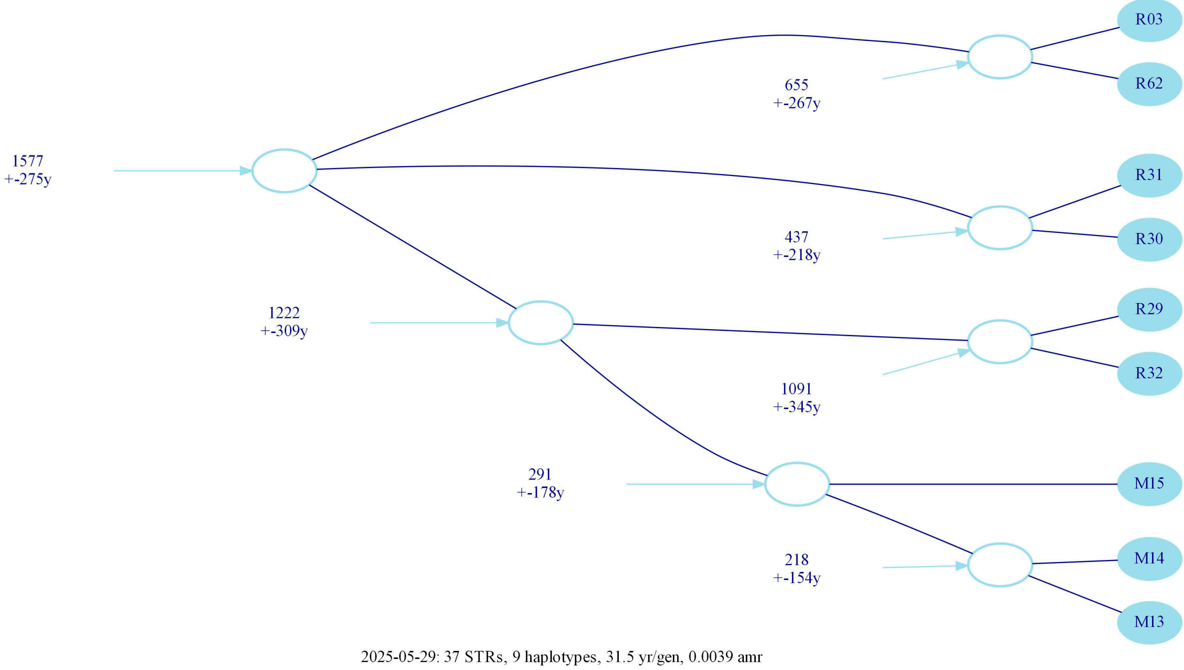

Supplementary Figure S9. Phylogenetic search tree for the branch R1a-Y2613 to confirm pre-Slavic origin of found Y-STR cluster. Designations of samples: R – Ryazan Russians; M – Moksha.

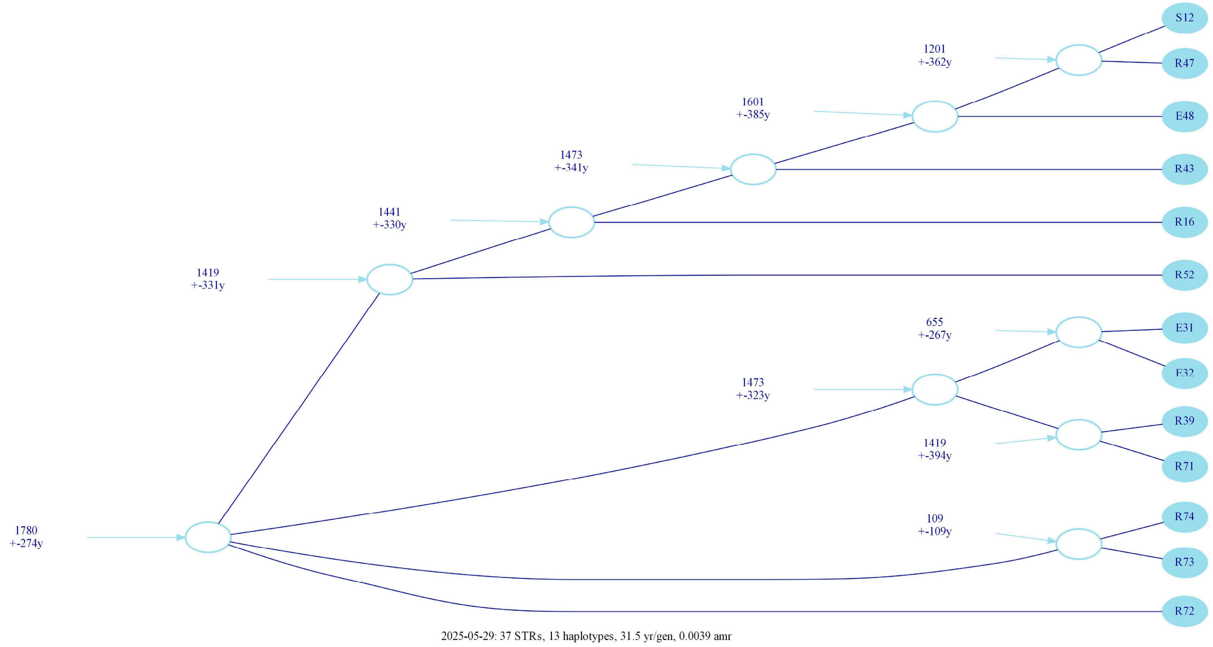

Supplementary Figure S10. Phylogenetic search tree for the branch R1a-S4481 to confirm pre-Slavic origin of found Y-STR cluster. Designations of samples: R – Ryazan Russians; E – Erzya; S – Shoksha.

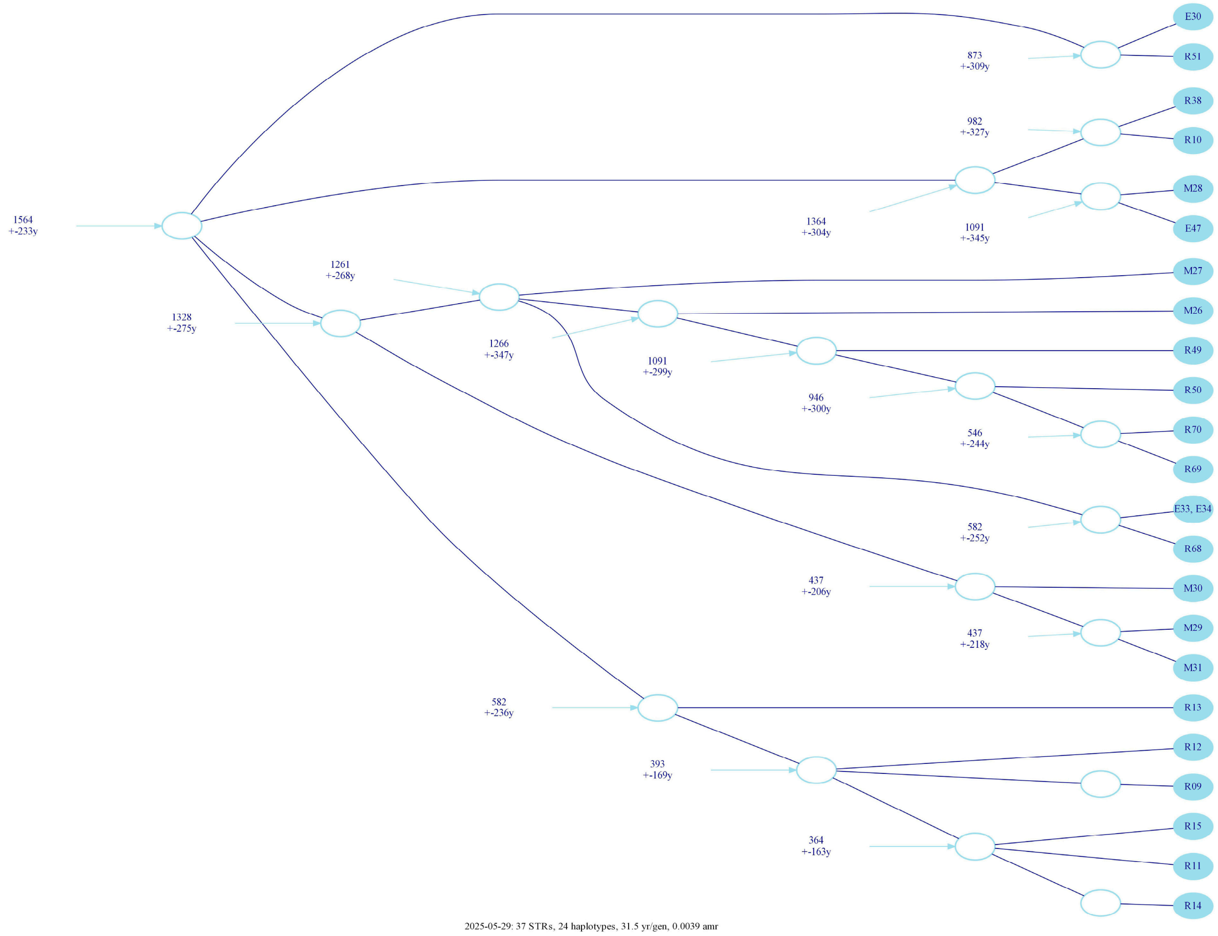

Supplementary Figure S11. Phylogenetic search tree for the branch R1a-YP683 to confirm pre-Slavic origin of found Y-STR cluster. Designations of samples: R – Ryazan Russians; E – Erzya; M – Moksha.

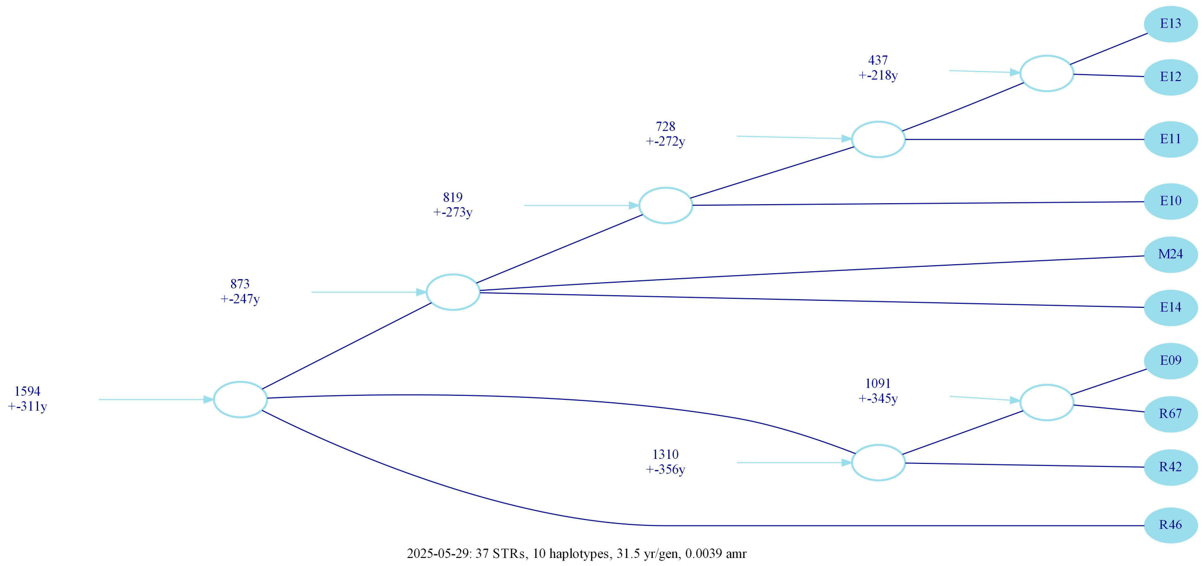

Supplementary Figure S12. Phylogenetic search tree for the branch R1a-Y11268 to confirm pre-Slavic origin of found Y-STR cluster. Designations of samples: R – Ryazan Russians; E – Erzya; M – Moksha.

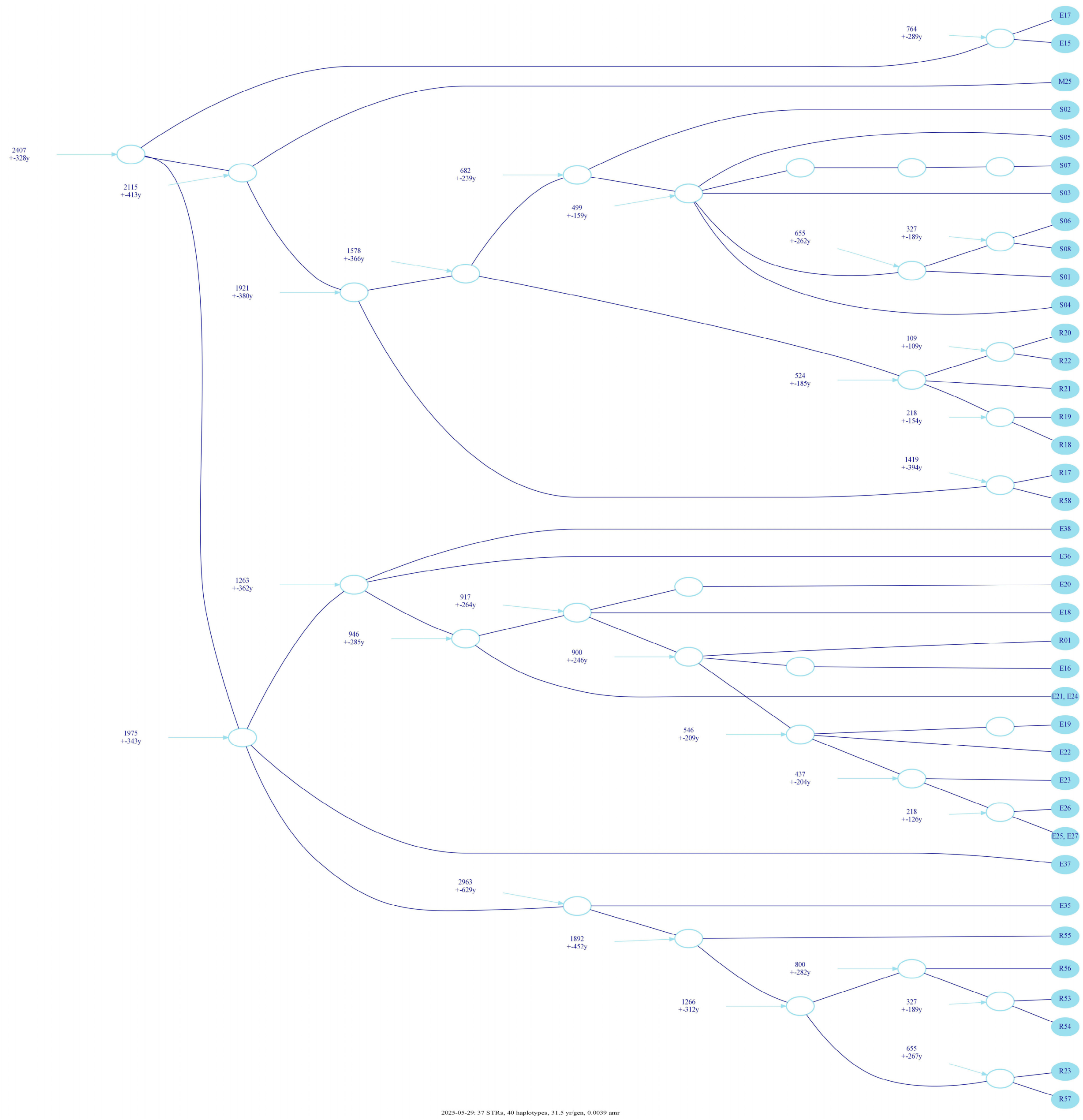

Supplementary Figure S13. Phylogenetic search tree for the branch R1a-Y33 (xY3301, Y1390) to confirm pre-Slavic origin of found Y-STR cluster. Designations of samples: R – Ryazan Russians; E – Erzya; M – Moksha; S – Shoksha.

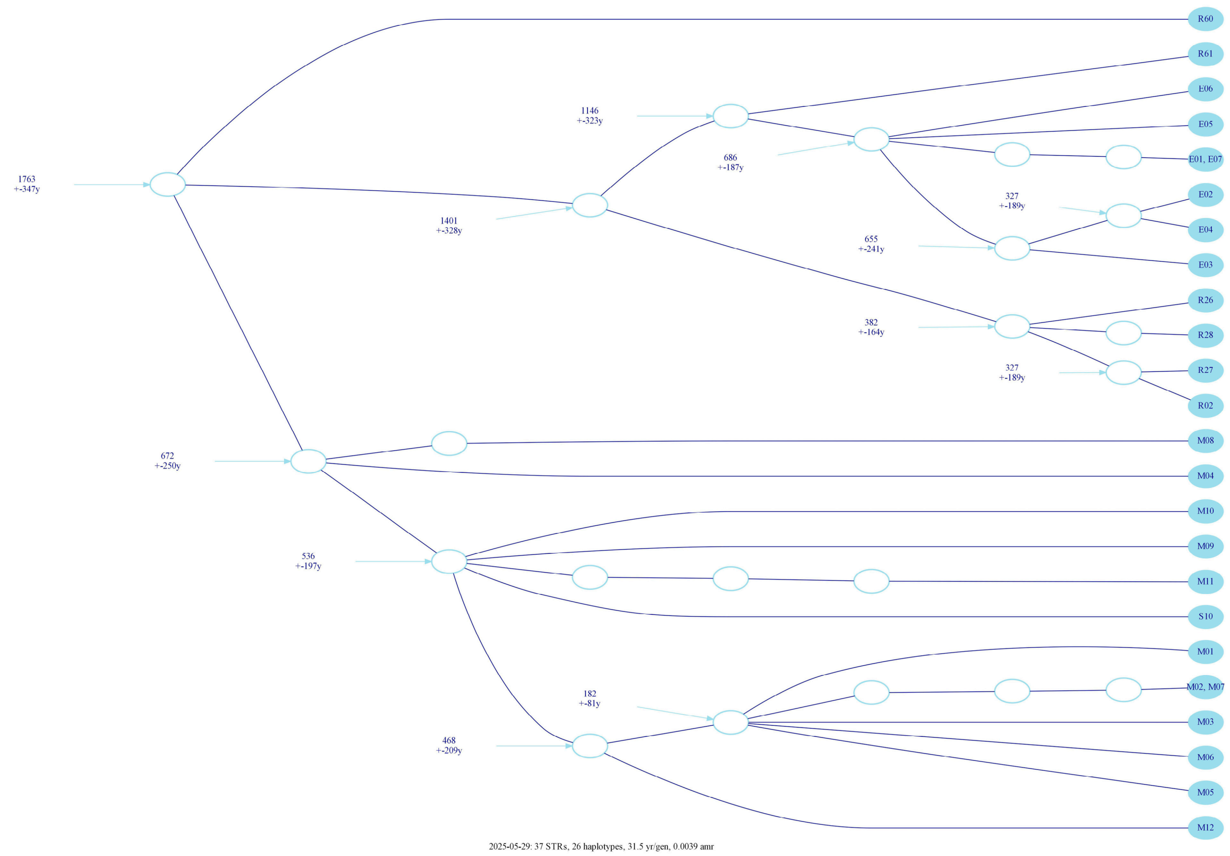

Supplementary Figure S14. Phylogenetic search tree for the branch R1a-YP304 to confirm pre-Slavic origin of found Y-STR cluster. Designations of samples: R – Ryazan Russians; E – Erzya; M – Moksha; S – Shoksha.

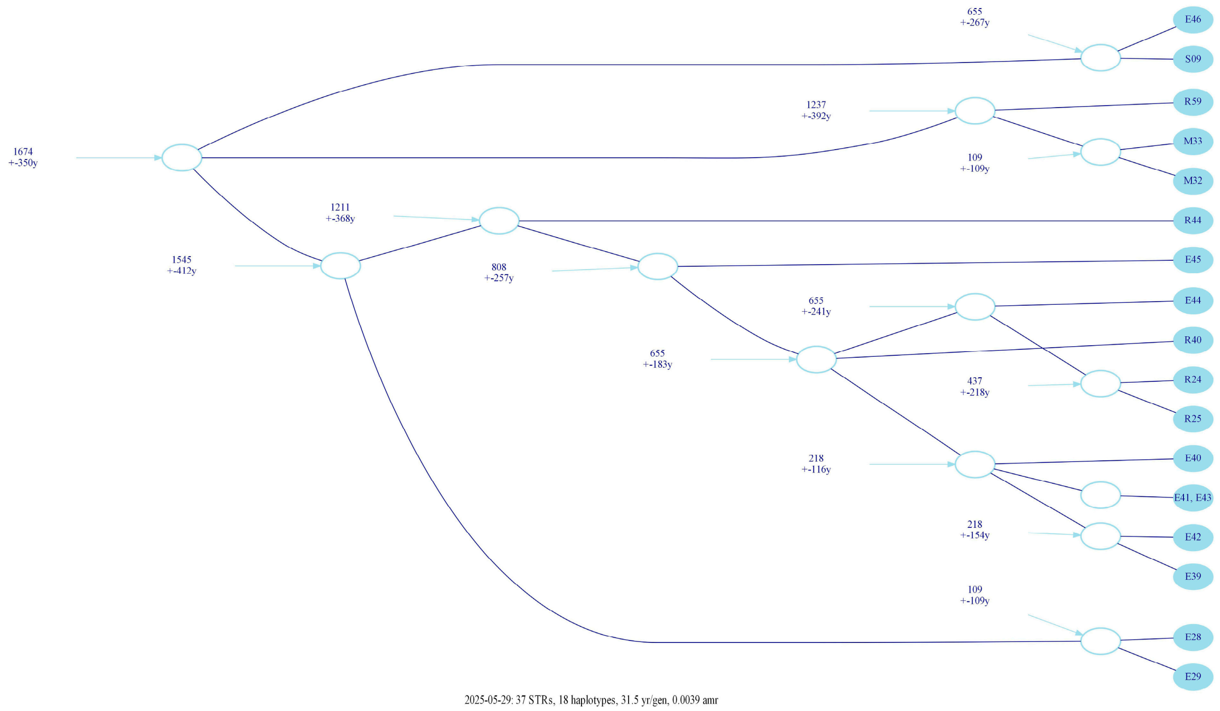

Supplementary Figure S15. Phylogenetic search tree for the branch R1a-Y2910 (xYP310) to confirm pre-Slavic origin of found Y-STR cluster. Designations of samples: R – Ryazan Russians; E – Erzya; M – Moksha; S – Shoksha.
